# Assessing the safety of microbiome perturbations

**DOI:** 10.1099/mgen.0.001405

**Published:** 2025-05-15

**Authors:** Aline Metris, Alan W. Walker, Alicia Showering, Andrea Doolan, Andrew J. McBain, Antonis Ampatzoglou, Barry Murphy, Catherine O'Neill, Colette Shortt, Elizabeth M. Darby, G Aldis, Greg G. Hillebrand, Helen L. Brown, Hilary P. Browne, Jay P. Tiesman, Joy Leng, Leo Lahti, Nicholas S. Jakubovics, Oliver Hasselwander, Robert D. Finn, Silvia Klamert, Tamas Korcsmaros, Lindsay J. Hall

**Affiliations:** 1 Unilever, Safety, Environmental and Regulatory Sciences (SERS), Sharnbrook, UK; 2 Microbiome, Food Innovation and Food Security Theme, Rowett Institute, University of Aberdeen, Aberdeen, UK; 3 BugBiome, Cambridge, UK; 4 Atlantia Clinical Trials, Cork, Ireland; 5 Division of Pharmacy and Optometry, School of Health Sciences, Faculty of Biology, Medicine and Health, The University of Manchester, Manchester, UK; 6 Unilever R&D Port Sunlight, Bebington, Wirral, UK; 7 Division of Dermatology and Musculoskeletal Sciences, Faculty of Biology, Medicine and Health, The University of Manchester, Manchester, UK; 8 Ulster University, Coleraine, NI BT52 1SA, Ireland; 9 Department of Microbes, Infection and Microbiomes, School of Infection, Inflammation and Immunology, College of Medicine and Health, University of Birmingham, Birmingham, UK; 10 Reckitt, Slough, UK; 11 University of Cincinnati, James L. Winkle College of Pharmacy, Cincinnati, OH, USA; 12 School of Biosciences, Sir Martin Evans Building, Cardiff University, Museum Avenue, Cardiff CF10 3AX, UK; 13 School of Microbiology, University College Cork, Cork, Ireland; 14 APC Microbiome Ireland, University College, Cork, Ireland; 15 The Procter & Gamble Company, Mason, OH, USA; 16 Institute of Infection, Veterinary and Ecological Sciences, University of Liverpool, Liverpool, UK; 17 Department of Computing, University of Turku, Turku FI-20014, Finland; 18 School of Dental Sciences, Faculty of Medical Sciences, Newcastle University, Newcastle upon Tyne, UK; 19 Health & Biosciences, IFF, c/o Danisco UK Ltd., Reigate RH2 9PW, UK; 20 European Molecular Biology Laboratory, European Bioinformatics Institute (EMBL-EBI), Wellcome Genome Campus, Hinxton, Cambridge, UK; 21 Food, Microbiomes and Health, Quadram Institute Bioscience, Norwich Research Park, Norwich, UK; 22 Division of Digestive Diseases, Imperial College London, London, UK; 23 NIHR Imperial BRC Organoid Facility, Imperial College London, London, UK

**Keywords:** artificial intelligence (AI)/ML, clinical studies, microbiome, oral, safety assessment, skin, gut, *in vitro* models

## Abstract

Everyday actions such as eating, tooth brushing or applying cosmetics inherently modulate our microbiome. Advances in sequencing technologies now facilitate detailed microbial profiling, driving intentional microbiome-targeted product development. Inspired by an academic-industry workshop held in January 2024, this review explores the oral, skin and gut microbiomes, focussing on the potential long-term implications of perturbations. Key challenges in microbiome safety assessment include confounding factors (ecological variability, host influences and external conditions like geography and diet) and biases from experimental measurements and bioinformatics analyses. The taxonomic composition of the microbiome has been associated with both health and disease, and perturbations like regular disruption of the dental biofilm are essential for preventing caries and inflammatory gum disease. However, further research is required to understand the potential long-term impacts of microbiome disturbances, particularly in vulnerable populations including infants. We propose that emerging technologies, such as omics technologies to characterize microbiome functions rather than taxa, leveraging artificial intelligence to interpret clinical study data and *in vitro* models to characterize and measure host–microbiome interaction endpoints, could all enhance the risk assessments. The workshop emphasized the importance of detailed documentation, transparency and openness in computational models to reduce uncertainties. Harmonisation of methods could help bridge regulatory gaps and streamline safety assessments but should remain flexible enough to allow innovation and technological advancements. Continued scientific collaboration and public engagement are critical for long-term microbiome monitoring, which is essential to advancing safety assessments of microbiome perturbations.

**Impact Statement**

Microbiome-targeting products are becoming increasingly prevalent, but there remains a significant need to develop a robust system for safety assessment. Our review, inspired by discussions from an academic-industry workshop, addresses the challenges of evaluating microbiome perturbations across the oral, skin and gut ecosystems. We highlight the complexity of linking microbiome changes to host outcomes and the variability introduced by external factors such as diet and drugs. We propose that while harmonisation of methods, data analysis and regulatory frameworks could advance microbiome risk assessment, it must account for the diversity of microbiome sites and populations, ensuring flexibility to adapt to evolving technologies and context-specific needs. Emerging technologies, such as *in vitro* models, artificial intelligence and metagenomics, also hold promise for enhancing safety evaluations, including in vulnerable populations (see Fig. 1). This review underscores the need for ongoing collaboration between academia, industry and regulatory bodies to develop robust and fit-for-purpose approaches for assessing microbiome-targeting products.

## Data Summary

All data associated with this work are reported within the article.

## Introduction

Human microbiomes are intimately linked to host health, disease, evolution and metabolic functions [1,3]. From birth, we are colonized by micro-organisms that form complex communities consisting of bacteria, phages, archaea and eukaryotes [4,5]. These microbiomes, present in various body sites such as the gut, skin, mouth and vagina, reflect highly evolved adaptations to their respective environments, which differ in structural composition, pH levels, nutrient availability, oxygen levels and immunological responses [6,7].

Early studies characterizing human microbiotas relied on 16S rRNA gene sequencing, which allowed taxonomic profiling primarily at the genus level by defining operational taxonomic units (OTUs) based on similarity thresholds. These initial studies revealed the diversity and compositional differences at different body sites and their stability (or lack thereof) over time [8,11]. Subsequent advancements in 16S rRNA gene analysis, such as amplicon sequence variants, and the development of metagenomic sequencing enabled higher taxonomic resolution to species or sub-species strain levels and functional pathway analysis indicative of health or disease states [7,12, 13]. Assembly-free approaches like 16S rRNA gene sequencing and metagenomic profiling rely on mapping sequence reads to reference databases containing sequences with assigned taxonomic information [14]. A limitation of these methods is the inability to classify reads absent from the reference databases. Recent years have seen the generation of large publicly available genome-sequenced culture collections, primarily from previously uncultured gut bacteria, improving metagenome reference databases by filling in some of the missing taxonomic information [15,18]. Metagenome-assembled genomes (MAGs) have further circumvented the need for genomes from cultured isolates, providing taxonomic and functional profiling without isolation [19,22]. Beyond sequence-only characterisations, technologies like metatranscriptomics, metabolomics and metaproteomics allow the profiling of active genes, metabolites and proteins, respectively [23,24].

While microbiome communities remain relatively stable over time in the absence of significant perturbations, the composition can shift day to day, influenced by external factors, particularly during early life when the microbiome is still developing. Birth is the first major transmission event, with a subset of commensal microbes transferring from mother to baby, with further environmental transmission continuing throughout life within family and social networks [25,28]. Human lifestyles, including diet, medications and healthcare products, also influence microbiota composition in different body sites over varying timescales [29,32]. In traditional risk assessment, the concept of ‘history of safe use’ has been applied to evaluate the safety of ingredients or products based on their long-standing use without adverse effects. Interventions such as cosmetics and fermented foods have long been utilized and modulate the microbiome, and their safety assessments can be based on extensive historical use and the absence of evidence of harm. However, advancements in microbiome science have led to the development of innovative products specifically designed to target the microbiome. These innovations, while promising, raise new potential safety implications that the concept of ‘history of safe use’ cannot fully address.

Microbiome-targeting products vary in composition and regulatory classifications depending on their intended use. They may include probiotics, prebiotics, postbiotics or combinations thereof and fall into categories such as food, cosmetics, medicines or drugs/biological products/live biotherapeutic products (LBPs). Notably, classification can be complex, and a product may fit into more than one category, and regulatory perspectives differ globally, with varying definitions and pre-market authorisation procedures which further complicate matters. The definitions used for this manuscript (not necessarily the ones used by the different regulatory bodies) are given in Table 1.

**Table 1.** Strategies to modulate the gut microbiome – risk-benefit and risk mitigation

Intervention	Definition	Health benefit	Associated risk	Uncertain risk	Risk mitigation
Faecal (intestinal) microbiota transfer [69]	Reconstitution of a perturbed microbiota with the beneficial stool microbiota of a healthy individual [157]	Therapy to prevent recurrent *C. difficile* infection [158,159] Potential therapy for other diseases associated with severe microbiota perturbation (though current evidence is less persuasive) [160]	Poorly screened samples may lead to infections, such as bacteraemia	Unknown long-term effects of administering these microbes, especially in children or young adults, potentially leading to disease later in life, e.g. obesity, colorectal cancer and inflammatory bowel disease	Rigorous donor and sample screening, including for antibiotic resistance genes; move towards use of defined combinations of gut microbes with similar efficacy as whole stool; optimize delivery to reduce risks such as aspiration pneumonia
Probiotics (including next-generation probiotics)	Live micro-organisms that, when administered in adequate amounts, confer a health benefit on the host [161]	May improve gut health, support regularity, prevent diarrhoea and reduce the risk of upper respiratory tract infections and eczema in children [162,163]	Traditional probiotics have been linked to rare fatal infections in immunocompromised patients; probiotics can translocate into the blood, though this is very rare	Lack of history of safe use for next-generation probiotics; unknown effects on gut community; potential for certain probiotics to metabolise drugs, altering their effectiveness	Rigorous strain characterisation (genotypic/phenotypic); *in vitro* and preclinical safety studies; systematic monitoring of adverse effects in clinical studies
Live-bacterial products (LBPs)	Single strain or defined consortia of bacterial strains designed to treat particular diseases	FDA approved for *C. difficile* infection; other products are in clinical trials for different indications including inflammatory bowel disease, such as ulcerative colitis, and improved response to checkpoint inhibitors for cancer treatment	Currently not well defined	Lack of history of safe use, so currently hard to define	Products will undergo clinical trials before coming to market
Postbiotics	A preparation of inanimate micro-organisms and/or their components that confer a health benefit on the host [35]	Emerging evidence suggests benefits for metabolic health [164]	Still emerging, so risks are not well-defined	Unknown long-term impacts due to the novelty of the intervention	Continued research and validation in both preclinical and clinical settings; low/no colonisation risk
Fibres and prebiotics (including human milk oligosaccharides)	Prebiotics: substrates selectively utilized by host micro-organisms that confer a health benefit [34,165]	Postulated benefits include promoting gut health, supporting regularity and reducing risks of cardiovascular and metabolic disease [163]	Risks may vary depending on individual microbiota; high-fibre diets may not be tolerated by some individuals	Possible increase of fibre-degrading species linked to diseases (e.g. *Prevotella/Segatella* and arthritis [166] or pathobionts	Microbiome profiling to ensure prebiotics/diet do not increase virulence or infection-associated members
Synbiotics [167]	A mixture comprising live micro-organisms and substrate(s) selectively utilized by host micro-organisms that confers a health benefit on the host		Combination of probiotics and prebiotics may lead to gastrointestinal discomfort, and imbalances in microbial strains or overuse in vulnerable populations could potentially cause adverse health effects	Combination of uncertain risks highlighted for probiotics and prebiotics	Like the probiotics above, they should be characterized to ensure safety, including a publicly available genome sequence and annotation assessed for any genes of safety concern (e.g. toxin production or transferrable antibiotic resistance)
Diet [168]	A specific selection of certain foods (e.g. Mediterranean diet) [168]	Reduced incidence of diseases, frailty and inflammation [169]	Variability in individual microbiome responses	Potentially unanticipated shifts in microbiota composition leading to off-target effects	Dietary recommendations based on individual microbiota profiles
Antibiotics	Chemicals that inhibit the growth of or kill bacteria [170]	Treat infections that could lead to death if untreated; prophylactic use to prevent infections	Off-target effects may reduce beneficial gut microbiota members across different body sites; disruption of the colonic mucosa barrier	Long-term reduction in beneficial microbes may contribute to disease development later in life	Use narrow-spectrum antibiotics where possible to minimize off-target effects

Globally, the Food and Agriculture Organization and World Health Organization (WHO) definition of probiotics – ‘live micro-organisms which when administered in adequate amounts confer a health benefit on the host’ – is widely accepted but inconsistently applied across different regions. To address this, a Codex Alimentarius Commission guideline proposal is currently under discussion, aiming to provide consistent terminology for probiotics [33]. Similarly, scientific definitions for prebiotics and postbiotics have been proposed to bring more clarity to their usage by the International Scientific Association for Probiotics and Prebiotics [34,35]. When it comes to regulating health claims in food, the EU follows a strict pre-market authorisation procedure, which includes specific risk reduction claims [36]. In contrast, the US regulatory system distinguishes between structure–function claims, which do not require pre-market authorisation, and health claims, which do [37,38]. This discrepancy underscores the need for clearer global alignment in this space. For cosmetic applications, the International Cooperation on Cosmetic Regulation is working towards harmonizing definitions for probiotics, prebiotics and postbiotics [39]. Both the EU and the USA allow the inclusion of live micro-organisms in cosmetic products, as reflected in databases like the Cosmetic ingredient database of the European Commission in Europe and similar regulations in the USA [40,42]. In the pharmaceutical arena, LBPs must meet stringent regulatory standards [43]. In the EU, they are regulated under the framework for biological medicinal products [36] and the European Pharmacopoeia [44]. Similarly, in the USA, the Food and Drug Administration (FDA) established guidelines for LBPs in 2012 and has since approved the first faecal microbiota therapy for recurrent *Clostridioides difficile* infection [45,46]. This demonstrates the evolving landscape for microbiome-based therapeutics as regulatory bodies work to adapt to innovations.

**Fig. 1.**
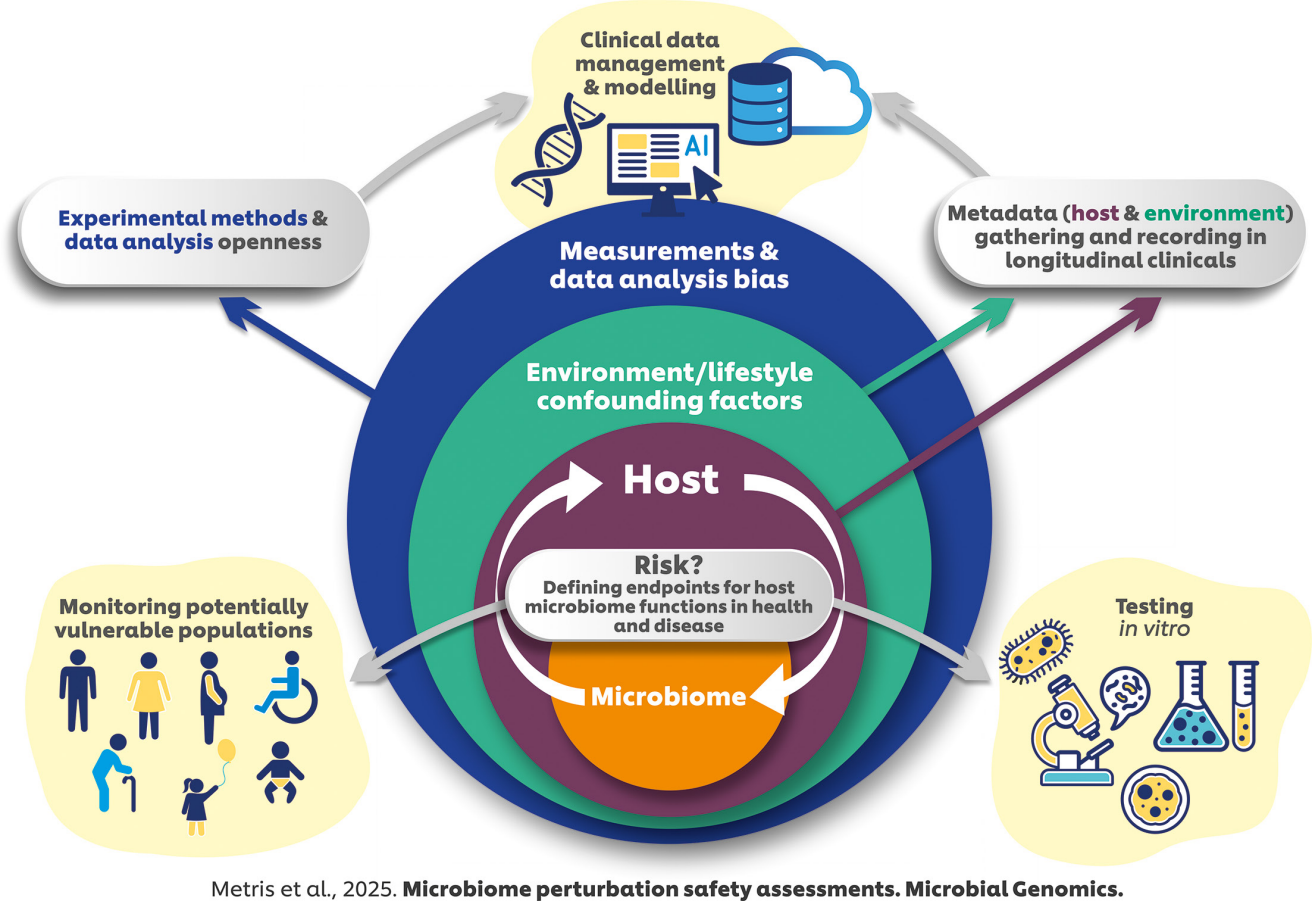
Summary of the workshop outputs. Endpoints defining host–microbiome interactions in health and disease remain poorly defined. Effective risk assessment is challenging due to uncertainties introduced by confounding factors such as lifestyle and environmental influences (green circle), as well as biases inherent in measurement techniques and data analysis methods (blue circle). To address these challenges, we propose integrating standardized *in vitro* testing and longitudinal monitoring of vulnerable populations to better assess potential risks. Additionally, uncertainties can be reduced by capturing extensive host metadata, utilizing advanced digital processing techniques, including emerging AI approaches and employing open access computational models.

Given advancements in microbiota profiling approaches such as metagenomics, increased interest in microbiome-targeting innovations and the lack of harmonisation in legislation, a joint academic-industry workshop was organized by the Microbiology Society from 24 to 25 January 2024. Participants from academia, industry and regulatory bodies with expertise ranging from microbiology to artificial intelligence (AI) discussed the potential medium- to long-term implications of perturbations in the gut, skin and oral microbiomes, and current research and developments required to assess these perturbations with respect to human health. In the first section of the manuscript, we explore the oral, skin and gut microbiomes, highlighting the evidence of their perturbations and associated health implications. The second section is focused on the design of longitudinal studies and how AI-driven approaches could enhance microbiome risk assessments. In the third section, we evaluate the potential as well as the limitations of *in vitro* systems for microbiome research. We end with a discussion of the challenges posed by current regulatory frameworks and the need for more adaptive approaches. The manuscript covers topics discussed at the workshop, with a particular focus on safety rather than efficacy. Other subjects, such as the use of animal models, the application of AI in risk assessment beyond addressing uncertainties about confounding factors and considerations for a new regulatory framework, were not covered in the workshop and are, therefore, not included. Given the broad scope of topics, the review is not exhaustive but aims to highlight seminal work and provide illustrative examples. The workshop outputs are summarized in Fig. 1.

## Part 1: Microbiome body sites, their importance in human health and the safety of their perturbations

Microbiomes inhabit distinct ecological niches within the human body, each shaped by unique environmental conditions and selective pressures. Understanding these individual environments and the factors influencing microbial composition is crucial for understanding the potential risks associated with altering/perturbing these microbial ecosystems with targeted microbiome interventions. Below, we explore three distinct human-associated microbiomes that represent the major ‘sites’, oral cavity, skin and gut, currently targeted via different intervention strategies and products and consider the evidence for potential long-term adverse effects.

### Gut microbiome

The gut microbiome is the most extensively studied microbial ecosystem, which includes a complex highly individualized community of micro-organisms in the digestive tract, playing key roles in numerous host responses including immune development, breakdown and metabolism of food and infection resistance [47,49]. During early life, the microbiome plays a pivotal role in infant development and is influenced by the maternal microbiota and diet, leading to a mature microbiota essential for health. *Bifidobacterium* represents the keystone early life microbiota genus, which is often dominant in vaginally delivered, breast-fed babies due to vertical transmission events and prebiotic effects of human milk oligosaccharides in maternal breast milk [50]. In adults, the gut microbiota composition is highly individualized, but with common dominant genera such as *Bacteroides*, *Faecalibacterium*, *Ruminococcus* and *Blautia* [51]. Studies have highlighted significant variations across populations, particularly between those in high-income and low- and middle-income countries [22,52,54]. Defining a ‘healthy’ gut microbiome remains challenging due to its variability influenced by factors like age, diet, lifestyle, geography, ethnicity, and medication use [55,56]. Previous studies have also suggested that resilience and stability are generally supported by high diversity and metabolic redundancy, which is why, when focusing on specific taxa, it is also useful to consider the metabolic functions present [55,57]. The gut microbiome has been indicated to influence a wide range of health and disease parameters, including those for inflammatory bowel diseases, colorectal cancer and cardiovascular and metabolic diseases [51]. Gut microbiome states have also been associated with long-term incident disease and mortality risk [58,59]. However, determining if gut microbiota perturbations cause or result from disease is challenging [60].

Strategies to manipulate the gut microbiome include dietary interventions, prebiotics, probiotics, antibiotics and faecal microbiota transplants (FMTs); see Table 1 for more details. While FMT is effective for preventing recurrent *C. difficile* infections, concerns remain regarding the transmission of infections and antibiotic-resistant organisms [61,62]. The safety and efficacy of traditional probiotics are generally well-recognized, though this is strain and condition-specific, with rare cases of serious infections also reported, particularly in immunocompromised patients [63,64]. Indeed, recent reports, as well as an FDA safety alert (2023), have highlighted the potential risks associated with probiotic use in neonatal intensive care settings, reinforcing the need for rigorous safety assessments and risk mitigation strategies in product development and clinical applications [65]. These instances raise important questions about whether adverse effects are primarily driven by the microbiome or the host’s response, underscoring the need for a deeper understanding of host–microbiome interactions. Additionally, there is a need to define clear and further endpoints for safety assessments to better evaluate the risks and benefits of probiotic use across different populations. Prebiotics and high-fibre diets can beneficially modulate the gut microbiome; however, they may also cause bloating and intestinal discomfort [66]. Moreover, the impact of antibiotics and other small molecular inhibitors on gut microbiota diversity and the host itself, along with the potential for prolonged disruptions and persistent antibiotic-resistant populations, underscores the need for careful assessment of such interventions (Table 1) [49,67, 68].

A significant challenge for the field lies in understanding the long-term implications of gut-targeted microbiome interventions. While most safety assessments focus on short-term effects, there is a growing recognition that microbiome alterations could have long-lasting impacts on health, potentially contributing to the development of autoimmune, neurological and metabolic disorders [69]. Ongoing research and updated regulations are essential for balancing the risks and benefits of microbiome-based therapies.

### Oral microbiome

The oral microbiota, colonizing the hard and soft tissues of the oral cavity, is essential for maintaining oral health. While salivary glands are sterile, saliva becomes heavily populated with micro-organisms upon entering the oral cavity, reaching concentrations of up to 10^9^ microbial cells per ml, predominantly attached to sloughed epithelial cells or present in aggregates [70,71]. Core microbes generally associated with oral health include *Streptococcus* species, particularly *Streptococcus mitis* and *Streptococcus sanguinis*, as well as *Veillonella*, *Actinomyces* and *Haemophilus* [72]. Dental biofilms, known as dental plaque, also rapidly form on tooth surfaces, and if not adequately managed, these biofilms can lead to dental caries and periodontitis, two of the most common human diseases [73]. Dental caries results from frequent sugar consumption, which leads to a detrimental biofilm enriched with species such as *Streptococcus mutans*, *Prevotella* spp. and *Leptotrichia* spp. [74]. Periodontitis, on the other hand, results from gingival inflammation caused by biofilms at the gum margins, with a microbiome shift favouring Gram-negative proteolytic species like *Porphyromonas gingivalis* and *Tannerella forsythia* [75]. The composition of oral microbiota is also increasingly recognized for its systemic health implications, influencing conditions that extend beyond the oral cavity. Emerging research suggests that shifts in the oral microbial community can serve as potential biomarkers for systemic diseases, such as cardiovascular conditions, diabetes and even respiratory infections. Additionally, the oral microbiome may play a direct role in mediating systemic inflammation, contributing to these broader health impacts [76,77]. Indeed, the oral microbiome may act as a reservoir for pathogens that can spread to other body sites, contributing to systemic infections and inflammatory conditions [78].

Notably, the resilience of the oral microbiome to short-term perturbations, such as changes in diet and oral hygiene practices, is considered indicative of oral health (Table 2) [79,80]. Current oral health recommendations, such as those from the WHO, emphasize regular oral hygiene practices, including twice-daily brushing with fluoride toothpaste, to reduce bacterial overgrowth of potential pathogens in the oral cavity [81,82]. Fluoride reduces the incidence of caries by supporting enamel remineralization, but it may also impact microbiota composition, increasing *Bacteroides* and reducing *Neisseria* and *Haemophilus* populations [83]. Additionally, the use of complementary strategies, used in combination with fluoride, has been explored, such as through biofilm disruption enhancing acid-neutralizing pathways which may further perturb the microbiome [81,84]. New approaches, such as enzyme-containing toothpaste and postbiotic applications in oral care products, have also been explored [31]. The long-term effects of interventions, particularly associated with antimicrobials and mouthwash use, are still being investigated. While some studies have raised concerns about the potential for increasing antibiotic-resistant bacteria, direct clinical evidence of harm remains inconclusive [85,87].

**Table 2.** Examples of longitudinal intervention studies, including randomized clinical trials, illustrating the effects of product use on the oral and skin microbiomes Oral hygiene interventions have been shown to shift the oral microbiome towards a healthier state compared to the absence of hygiene (as seen in experimental gingivitis studies). Similarly, cosmetic products impact the skin microbiome, with effects varying by body site but they are typically less significant than interpersonal variation. Certain antimicrobials, such as ethanol and povidone iodine, induce short-term, reversible alterations in the forearm skin microbiome. Notably, current studies disproportionately represent populations from Western countries and China, highlighting a gap in global microbiome research coverage (for recent comprehensive reviews, see [171,172]).

	Product type	Study design			Microbiome measurement		Reported outcome	
		Population/size	Regimen	Test vs. control	Site	Technique	Diversity analysis and qPCR measurements	Composition (relative abundance)
Oral microbioe	Fluoride toothpaste containing enzymes [31]	Healthy, over 18 years old, 111 subjects completed, UK	Brushing twice a day for 14 weeks	Fluoride toothpaste (1,450 ppm) vs. fluoride toothpaste containing enzymes and proteins (Zendium™)	Supragingival dental plaque	16S rRNA gene sequencing (V4–V6 region)	na	Statistical analysis shows significant increases in 12 taxa associated with gum health including *Neisseria* spp. and a significant decrease in 10 taxa associated with periodontal disease including *Treponema* spp.
	Fluoride and arginine-containing dentifrices [84]	53 patients, age≥16 years old	Baseline after a 1-week washout period to brush 2×/day with a fluoride-containing dentifrice, 3 months after use of a 1,450-ppm fluoride dentifrice and 6 months after using a 1,450-ppm fluoride with 1.5% arginine dentifrice	Caries active (*n*=26) vs. caries free (*n*=27)	Supragingival dental plaque	16S rRNA gene sequencing (V4–V6 region), shotgun and meta-transcriptomics	Unaffected	After the 3 weeks of fluoride tooth brushing, several caries-associated bacteria were reduced, and there was also an increase in several health- and periodontitis-associated bacteria in both caries and caries-free sites. After the fluoride+arginine dentifrice, there was a further decrease of both caries- and periodontitis-associated organisms, and a decrease of genes from the arginine biosynthesis pathway was also observed, in addition to an increase in the expression of genes associated with the arginine deiminase pathway
	Stannous fluoride toothpaste [173]	Healthy non-smokers over 18 years old, Canada	Preinduction phase of 1 weeks with brush twice daily with a soft manual toothbrush using control dentifrice followed by use of test or control products for 2 weeks, and from day 0, 3 weeks abstaining from all oral hygiene practices followed by dental prophylaxis (day 21) and brushing with test or control products was reinstituted for 3 weeks (day 42)	Control group: (*n*=17) sodium monofluorophosphate 0.76% toothpaste vs. test group (*n*=16) zinc phosphate toothpaste	Supragingival dental plaque	16S rRNA gene sequencing (V3–V4 region)	Increase of Shannon diversity with abstaining of hygiene practice (both groups); test group has a lower diversity at 0 and day 42 than the control group	*Bacteroidales* as well as *Bacteroidota* members including *Porphyromonas* and *Tannerella* were impacted by the SnF2 treatment (different abundance after gingivitis resolution at day 42); a similar trend was observed at the species level for *Porphyromonas endodentalis* and *Tannerella forsythia*, as well as in other known oral pathogens, specifically within the *Treponema* genus, which includes species that are highly associated with red complex and periodontitis
	Chlorhexidine mouthwash [174]	Healthy non-smoker adults, UK	10 ml of mouthwash rinse for 1 min, twice/day for 7 days after brushing with a standardized toothpaste	Mouthwash: placebo (*n*=36) vs. 0.2% chlorhexidine (*n*=36)	Non-stimulated saliva	16S rRNA gene sequencing (V1–V2 region)	Chlorhexidine promoted reductions in Shannon diversity	Increases in the abundance of some genera *Escherichia*, *Hylemonella*, *Capnocytophaga*, *Granulicatella*, *Streptococcus* and *Neisseria* and decreases in *Prevotella*, *Actinomyces*, *Fusobacterium*, *Megasphaera*, *Campylobacter*, *Lachnoanaerobaculum*, *Catonella*, *Corynebacterium*, *Clostridium* and *TG5* with chlorhexidine compared to the placebo
	Cetylpyridinium chloride mouthwash [175]	Healthy 18 to 53 years old, China	After 3 weeks of standard regimen and professional cleaning, subjects refrained from mechanical oral hygiene and rinsed twice/day with 20 ml mouthwash for 30 s, for 21 days	Cetylpyridinium chloride (CPC) (*n*=56) vs. water (*n*=36)	Supragingival plaque	16S rRNA gene sequencing (V1–V3 region)	Αlpha-diversity in the control group exhibited a significant increase from baseline to day 21, whereas that in the CPC group remained stable	After 3 weeks, decrease of 17 with CPC use compared to water, including *Porphyromonas*, *Peptostreptococcus*, *Prevotella*, *Peptococcus*, *Selenomonas*, *Solobacterium*, *SR1*, *Tannerella*, *TM7 genus*, *Uncultured_Lachnospiraceae Atopobium*, *Gemella*, *Megasphaera*, *Mogibacterium*, *Moraxella, Oribacterium* and *Shuttleworthia*; increase of *Haemophilus* and *Lautropia* and *Neisseria*, *Capnocytophaga* and *Propionibacterium*
	CPC and essential oils mouthwash [176]	Healthy subjects: age 18–60 years; a minimum 20, USA	At baseline, within 2 weeks of first visit, dental prophylaxis and allocated into groups; refraining from brushing, rinsing was performed twice daily with 20 ml of the assigned mouthrinse; sample collection after 6 and 12 weeks	Control group *n*=41 (water rinse) vs. test *n*=39 (CPC+essential oil)	Supragingival plaque	16S rRNA gene sequencing (V1–V3 region)	Observed and Shannon diversity increased in both groups, although not significant; weighted UniFrac distances in composition were different between baseline and week 12 in the test group	33 OTUs at the end of the study that were differentially depleted in the treatment group compared to the control group included *Corynebacterium matruchotii*, *Corynebacterium durum*, *several Actinomyces, Fusobacterium, Leptotrichia, Capnocytophaga, Neisseria, Streptococcus, Aggregatibacter, Porphyromonas, Terrahaemophilus aromaticivorans* and *Lautropia*; 40 OTUs were overrepresented in the treatment group at week 12
Skin microbiome	Skin care products (body wash, moisturizer, sunscreen, antiperspirant and soothing foot powder) [32]	12 healthy individuals, USA	No use of any personal care product for weeks 1–3 except a mild body wash; during weeks 4–6, in addition to the body wash, participants were asked to apply selected commercial skin care products at specific body parts; weeks 7–9, return to their normal routine by using the same personal care products as prior to the study; samples collected once a week for most participants	Test products: a moisturizer on the forearm, a sunscreen on the face, an antiperspirant on the armpits and a soothing powder on the foot	Front of the elbow and front forearm, the upper cheek bone and lower jaw, armpits, between the first and second toe and between the third and fourth toe of the foot	16S rRNA gene sequencing (V4 region)	Higher Shannon diversity in arms and face at baseline for both female and male; refraining from using beauty products leads to a significant decrease in armpits and feet; a higher diversity was observed for armpits and feet of all individuals during the use of antiperspirant and foot powder, followed by a bacterial diversity decrease in the armpits when their regular personal beauty product use was resumed	Although the microbiome was site-specific, it varied more between individuals, and this inter-individual variability was maintained over time despite the changes in personal care routine; significant increase in abundance of Gram-negative bacteria *Acinetobacter* and *Paracoccus* genera for the armpits and feet of both females and males during the use of antiperspirant, while their abundance remained stable for the arms and face during that time; decrease in abundance of *Enhydrobacter* in the armpits of males; *Cyanobacteria*, potentially originating from plant material, also increased during beauty product use, especially in males, in the armpits and face of females and males
	Skin cleansing (soaps) [98]	10 healthy subjects, USA	0.5 ml of product was spread over the volar forearm with a gloved hand for 30 s and then rinsed off with tap water while being scrubbed with a gloved hand for 60 s; 16S samples were taken prewash, 10 min after, 24 h after and 3 days after washing; quantitative PCR (qPCR) samples prewash, after 10 min, 6 h and 24 h	Tests with soft soaps: soap A, lavender and chamomile hand soap; soap B, soothing aloe vera moisturizing hand soap; soap C, rich shea butter moisturizing hand soap; soap D, aquarium series hand soap; soap E, containing benzalkonium chloride; soap F, containing triclocarban vs. water	Forearm	16S rRNA gene sequencing (V1–V3 region), real-time qPCR for total bacteria and *Staphylococcus epidermidis*	No significant difference in the alpha-diversity was detected between soap D and water application at any sample time; no significant difference in total microbial abundance as measured by real-time qPCR in 24 h; the antimicrobial compounds benzalkonium chloride and triclocarban did not result in a statistically significant difference in abundance of *Staphylococcus epidermidis* at any sampling time	No difference in the taxonomic composition of the skin bacterial community between washing with soap D and water
	Ethanol [101]	10 healthy adults, UK	The skin area was cleaned using four 70% ethanol-soaked cotton wool pads (two for the volar and two for the dorsal) forearm by wiping; samples were taken post-wash after drying and at different times (2, 4, 6, and 24 h after wiping) at three different visits	70% ethanol	Volar and dorsal forearm	16S rRNA gene sequencing (V1–V2 region), qPCR for total bacteria and *Staphylococcus epidermidis*	Alpha-diversity (observed and Shannon diversity) was systematically higher for females than males and decreased with treatment for both males and females and recovered in 6 h; total eubacterial 16S decreased following ethanol wiping and recovered in 6 h; similar trend for *Staphylococcus epidermidis*, but recovery time was less obvious	Baseline samples taken for each individual at the three sampling visits indicate stability across time, and volar and dorsal sample profiles were highly similar within individuals; the wiping had little effect on relative composition depending on individuals
	Antiseptic agent [177]	13 healthy subjects over 21 years old, USA	A 1-in^2^ area was swabbed vigorously with ten swipes followed by ten additional swipes in the perpendicular direction before being administered one of four treatments for 1.5 min, using gentle swiping with a cotton pad soaked in 5 ml of the test agent; cotton pads and test agents were treated with UV for 20 min prior to use; sampling at baseline and after 1, 6, 12, 24, 36 and 72 h of administration	Alcohol (80% ethanol) vs. water (UltraPure Distilled Water, Invitrogen) – first visit and ovidone-iodine (10%) vs. chlorhexidine (chlorhexidine-gluconate 4%, results not analysed) – second visit	Volar forearm and the upper back	16S rRNA gene sequencing (V1–V3 region), qPCR for total bacteria	Alpha-diversity (Shannon diversity, observed species and equitability) was significantly higher on the forearm compared to back; beta-diversity (UniFrac) indicated that interpersonal variability and site specificity were the most significant contributors to variation; water, alcohol and povidone-iodine all significantly reduced the number of observed species on the forearm compared to adjacent controls at 6 h; water and alcohol were found to decrease overall bacterial load at each body site	Back communities were dominated by *Propionibacteriaceae* and *Staphylococcaceae,* while forearm hosted additional taxa, including *Streptococcaceae* and *Corynebacteriaceae*; accounting for interpersonal variability, lowly abundant members of the skin microbiota were more likely to be displaced and subsequently replaced by the most abundant taxa prior to treatment, while members of the skin commensal family. *Propionibactericeae* were particularly resilient to treatment

A critical future direction in oral microbiome research involves intensive sampling of diverse populations and conducting longitudinal studies using appropriate sampling methods, such as flocked swabs and saliva collection, to provide a comprehensive overview of the human oral microbiome over the host lifespan. These studies are essential for identifying early microbial biomarkers of diseases like dental caries and periodontitis. Additionally, investigations using clinical methods of experimental gingivitis could enhance risk assessment by enabling more precise predictions of how microbiome perturbations may impact oral health and by identifying factors that contribute to resilience. Developing predictive models of oral health, particularly those based on early-life microbiome analysis, remains a significant challenge. This is especially true when accounting for genetic and functional biomarkers, which go beyond traditional taxonomic units and may offer deeper insights into individual susceptibility to oral diseases.

### Skin microbiome

The skin microbiota consists of a diverse array of micro-organisms adapted to the skin’s unique environment, playing a fundamental role in maintaining skin health and barrier function [88]. The composition of the skin microbiota varies significantly across different body sites, influenced by factors such as moisture, sebum content and environmental exposure [89]. Key microbial residents include *Staphylococcus epidermidis*, *Cutibacterium acnes* and *Corynebacterium* spp. These populations shift across different life stages, with *Streptococcus* spp. abundant during early life, and *Cutibacterium acnes* becomes more prevalent during puberty due to increased sebum production, while *Staphylococcus* and *Corynebacterium* species dominate at other times [90,92]. The skin microbiome plays a crucial role in protecting against pathogenic infections and modulating immune responses. For instance, *Staphylococcus epidermidis* has been shown to produce antimicrobial peptides that inhibit pathogenic strains, thus contributing to skin defence [93]. However, perturbations in the skin microbiome have been associated with conditions such as acne, eczema and psoriasis and are thought to influence systemic inflammatory responses, potentially impacting overall immune health [94,95].

Longitudinal studies have demonstrated that the skin microbiome, even at the strain level, exhibits stability over months to years in healthy individuals [96,97]. Indeed, microbiome temporal stability and resilience to mild perturbations are hallmarks of healthy skin [98,100]. This stability is attributed to protected niches, such as hair follicles, skin invaginations and sweat ducts, which serve as reservoirs for microbes, enabling rapid recolonisation of the skin surface [99]. Frequent washing and the use of skincare products have the potential to influence the delicate balance of the skin microbiota. While harsh soaps, antiseptics and other cosmetic products can alter the skin’s pH and moisture levels, the direct impact on the microbial community and its implications for skin health remain an area of ongoing research (Table 2). Although concerns have been raised about potential disruptions leading to conditions such as dryness, irritation or increased susceptibility to infections, the current evidence linking these effects directly to changes in the skin microbiome is still limited and inconclusive [98,100,102].

Interventions targeting the skin microbiome, such as the use of probiotics, prebiotics, postbiotics and synbiotics (see Table 1 for definitions of these terms), are designed to modulate the microbial community. While probiotics can temporarily influence the composition of the skin microbiome, the ability to engraft – where introduced microbes establish themselves and persist long-term – appears to be rare [103,104]. This suggests that significant long-term alterations to the skin microbiome are unlikely, thereby reducing concerns about potential lasting effects from these interventions. Postbiotics, including non-viable bacteria and lysates, and prebiotics, such as lipids and amino acids mimicking sebum and sweat, offer other avenues for modulating the skin microbiome [105]. The combination of live commensals with physiological energy sources (i.e. synbiotics) may further improve engraftment rates and efficacy.

Future research should focus on elucidating the mechanisms underlying the stability and resilience of the skin microbiome, particularly in response to various treatments and environmental changes [106]. Additionally, there is a need for ongoing monitoring to assess the potential long-term effects of cosmetic and therapeutic products on skin microbiota diversity and composition, to confirm the absence of adverse effects.

**Summary Box 1: Common features and differences of the oral, skin and gut microbiomes and safety assessment of their perturbations.**

The human gut, skin and oral microbiotas are all diverse communities that play crucial roles in both disease and health. However, each microbiota’s composition is influenced by distinct biogeographies within different microenvironments [178]. For instance, the oral microbiome includes diverse habitats such as the teeth and soft palate, the skin microbiome varies across sebaceous (oily) and dry skin regions and the gut microbiome spans environments from the acidic stomach (pH 1.5–3) to the slightly acidic large intestine (pH 5.5–7). Common phyla across these three sites include *Bacillota* (formerly *Firmicutes*), *Pseudomonadota* (formerly *Proteobacteria*), *Actinomycetota* (formerly *Actinobacteria*) and *Bacteroidota* (formerly *Bacteroidetes*), though different subsets of constituent species tend to dominate each body site [179]. For example, *Bacillota* and *Bacteroidota* are prevalent in the gut and oral cavity, while *Actinomycetota* (also dominant in the gut of breast-fed infants) and *Pseudomonadota* are commonly found in the oral cavity and on the skin. All three body sites are targets for microbiome-modulating interventions including therapies aimed at improving or maintaining health. The skin microbiome is influenced by the daily use of cosmetics and skincare products, the gut microbiome is modulated by diet and medications and the oral microbiome is affected by diet and oral hygiene practices. Unlike the gut microbiome, the oral microbiome requires active modulation to maintain health throughout the life course; without regular interventions such as toothbrushing – while consuming a modern diet – individuals may develop dental caries and periodontitis [180]. The skin microbiome is inherently resilient to perturbations. Although cases of systemic infections linked to gut probiotics in vulnerable populations have been very rare, there is a need for ongoing monitoring to assess potential long-term safety implications. This monitoring may benefit from public involvement, such as the public reporting of adverse effects, consumer feedback on the effectiveness and tolerability of products and broader engagement in participatory health monitoring platforms. Involvement from the public can also help identify patterns of adverse reactions more rapidly and provide real-world data that may otherwise be missed in clinical trials, thereby improving post-market surveillance and safety assessments. Despite the differences among human microbiomes, a fundamental challenge in evaluating the safety of microbiome perturbations across all types is the presence of confounding factors and the lack of established biomarkers that clearly differentiate between health and disease.

## Part 2: Optimizing metagenomic clinical study design and recommendations, potential and limitations of AI Integration

Taxonomic analysis of the microbiota in clinical studies based on metagenomics has revealed associations between composition and health and disease, but there is a significant interindividual variation in microbiota composition, complicating efforts to define a ‘healthy’ microbiome and making regulatory and safety assessments challenging [56]. Indeed, longitudinal sampling plays a crucial role in microbiome research as it helps control for individual variability, allowing for the tracking of within-host changes over time rather than relying solely on cross-sectional comparisons, which may be confounded by inter-individual differences. Furthermore, given the absence of a universal definition of health, monitoring microbiome changes over time provides a more informative approach to identifying patterns associated with disease progression or resilience. This temporal perspective enhances our ability to infer causality by linking microbial shifts to specific physiological or environmental changes rather than relying on single time-point associations. Moreover, longitudinal samples would allow consideration of microbial engraftment – whether transient or permanent – as part of safety assessments, given its potential implications for long-term microbiota composition and host health outcomes [107]. So far, although promising, AI methods have shown mixed results in predicting health outcomes or identifying causal impacts of microbiome variations due to the inherent complexity and individual variability of these ecosystems [108]. We explored how effective clinical design complemented by developments in AI could help address these gaps.

### Powering and designing longitudinal studies

Longitudinal studies, particularly when synthesized through meta-analyses, offer evidence of the highest relevance and are a cornerstone for microbiome safety research. As the volume and diversity of microbiome data in public repositories grow (e.g. European Nucleotide Archive, MGnify), AI methods are increasingly used to integrate data from various cohorts, enhancing statistical power [109]. However, clinical studies must be meticulously planned and executed with well-defined objectives and robust study designs. A critical aspect of individual studies is ensuring they are sufficiently powered to detect subtle but significant microbiome perturbations [110]. Safety is a paramount concern in these studies, making it essential to rely on objective biomarkers as well as self-reported symptoms, which can be subjective, to provide a more comprehensive assessment. Randomized controlled trials (RCTs) are the gold standard for clinical study design, ideally conducted in a double-blind and placebo-controlled manner to eliminate bias. However, these can be very costly and complicated to set up. Cross-over designs, where each participant serves as their control, offer an advantage by minimizing interindividual variability in microbiota composition. Prospective studies can allow the detection of risk factors with epidemiological and public health relevance [58]. Ideally, comprehensive time-series data would help both measuring and understanding resilience and finding biomarkers of health and disease but are often challenging to obtain due to practical and ethical constraints. AI can prioritize targets for further study and predict the impacts of microbiome alterations, helping to design experiments that maximize the accuracy and relevance of findings [111]. Studies should include, at a minimum, baseline and end-of-intervention sampling points. It has been proposed that an additional sampling point at an appropriate time after the intervention to check the ability of the microbiome to return to its initial state could be used as a proxy for showing that microbiome resilience has not been compromised [110].

### Participant representativeness, intervention strategies and metadata considerations

**Fig. 2.**
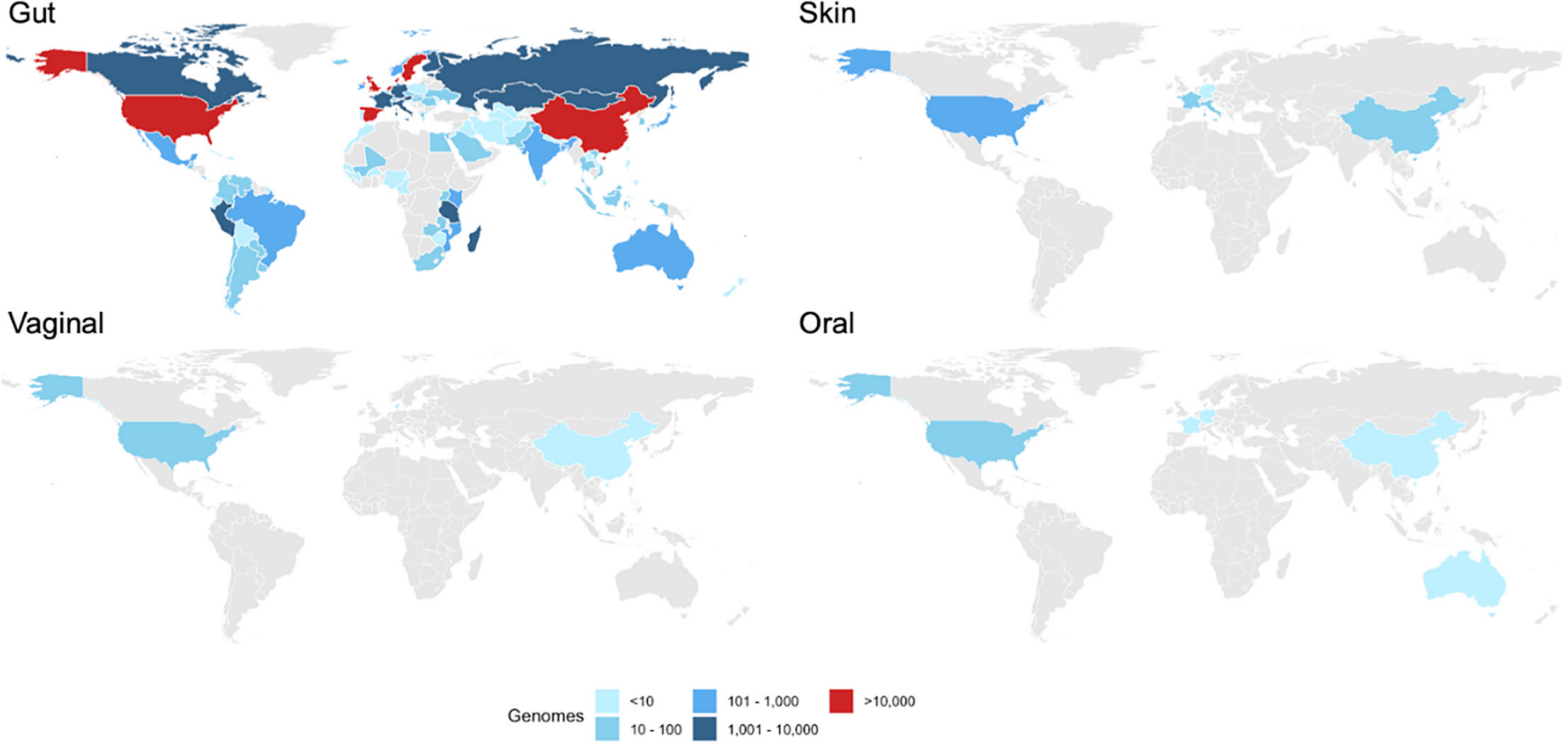
The distribution of genomes found in the four MGnify human microbiome catalogues: gut, skin, vaginal and oral. While each of these catalogues contains different numbers of genomes and is not directly comparable, the maps highlight the geographic skew of the samples contributing to the genomes found within these catalogues, with human microbiomes from North America, Europe and China representing the bulk of the data [181].

The selection of study participants must also be approached with care, incorporating relevant inclusion and exclusion criteria. Key factors include age, gender, health status, medication use, co-morbidities, diet and lifestyle. Additionally, the geographical location and environment (urban vs. rural) can significantly impact the microbiome and must be considered. For example, extensive catalogues of genomes from cultured microbes and MAGs have identified over 200,000 distinct genomes from tens of thousands of metagenomic datasets, representing ~5,000 distinct species in the human gut [20]. However, these samples predominantly come from North American, European and Chinese populations, highlighting a lack of global representation [112,114] (Fig. 2). Furthermore, these studies primarily focus on adult microbiotas, with less attention given to the microbiotas of infants and the elderly, which have distinct compositions. The microbiomes of other body sites, like the skin, vagina and oral cavity, are underrepresented in global studies, despite their accessibility and importance. The adaptability of AI allows for the characterisation of these microbiomes with high accuracy, though generalizability to new samples and populations is limited by geographic and demographic variations in available data. Baseline microbiota composition is also a critical variable that can influence an individual’s response to an intervention, necessitating careful consideration during participant selection [66]. Registry-based studies can offer highly standardized individual-level data on a population scale, with a strong research tradition, particularly in Nordic countries [115]. However, access to these data for research purposes can vary significantly between countries, depending on national regulations and data-sharing policies. More broadly, inclusive research is required to understand the full scope of microbiome variations and their implications on host health [116].

The investigation products or interventions used in these studies must be meticulously characterized, which includes verifying the intended dose for realistic exposure and ensuring safety concerning microbiological or chemical hazards. Studies that test multiple doses, possibly in a cross-over manner, can provide valuable insights into dose–response relationships, while initial exploratory studies, including single-arm studies, may be required to design subsequent RCTs effectively [117]. Factors such as host genetics and environmental influences, which are often not recorded in databases due to privacy concerns and cost, further complicate these analyses. Non-linear relationships, like dose-dependent effects and individual responses influenced by microbiota composition or host immune interactions, are common and significant for safety evaluations. AI can help identify hidden confounders and facilitate automated biomarker discovery, though the complexity of these relationships often exceeds the capabilities of available data [108].

### Methods and data bias

Sampling strategies are also key and must be tailored to the specific microbiome under investigation. For instance, skin microbiome studies might use punch biopsies, tape strips, cup-scrubs or swabs, while oral microbiome studies may sample from soft tissues like the tongue or hard palate, or hard tissues such as dental plaque, and faecal samples are commonly used as proxies for gut mucosal microbiota instead of biopsies [118,120]. Standardized sample collection practices could help with reproducibility but come with their own set of challenges. It’s important to acknowledge that no single protocol is without bias, and the inherent variability in microbial communities across individuals complicates the application of one-size-fits-all methodologies. Protocol choices, such as DNA extraction techniques or sample preservation methods, can introduce biases that influence study outcomes, particularly when comparing diverse populations or microbial compositions (e.g. differences in Gram-positive vs. Gram-negative bacterial lysis) [60]. Rather than strict standardisation of sampling protocols, the emphasis should be on transparent documentation of methods and thoughtful selection of protocols tailored to the study’s specific context. This would allow for better interpretation of results within and across studies while acknowledging the limitations imposed by different techniques. Other aspects such as defining the exact biogeography for skin samples, for example [121], metadata standards and reporting requirements, can mitigate some sources of bias and facilitate data comparison across studies while taking into account the evolving scientific understanding and specific study needs. Researchers must also weigh logistical and ethical considerations, balancing scientific rigour against participant burden and available resources. Flexibility in adapting methodologies based on the study population and microbial composition is essential to maintaining both relevance and validity in microbiome research, particularly in longitudinal or population-based studies.

The choice of bioinformatics tools and reference databases also adds to the experimental bias [122,123]. The downstream analysis of microbiome data should, therefore, encompass a range of endpoints, including (but not limited to) alpha- and beta-diversity, taxa richness, the relative abundance of specific taxa and functional activity. However, determining which microbiome features are most informative for applications like diagnostics or safety assessments remains unclear. In addition, the methods used to estimate these features and whether they are considered in absolute terms or relative abundance, which require compositional analysis, can also introduce some bias [124,125].

The application of AI in microbiome safety assessments is challenging due to inconsistent reporting of metadata, such as participants’ demographics and the experimental conditions [126]. Nonetheless, even with limited metadata, exploratory analyses can still yield valuable insights, especially when integrated with more comprehensive data sets. While the lack of consistent, high-quality metadata hampers progress, AI tools like text mining and large language models can augment datasets by inferring missing information and suggesting potential functions or interactions [109]. Nonetheless, differences in reference databases and analytical tools can lead to inconsistent results between studies, complicating comparisons. Open access to research data and methodologies promotes greater transparency and reproducibility, which plays a vital role in reducing bias and ensuring more robust, reliable outcomes across studies [127]. Discussions at the workshop emphasized the limitations of current AI-driven tools and the need for rigorous validation and implementation strategies before they can be reliably integrated into regulatory decision-making. Biases related to data representativeness and patient attrition are common challenges, and even advanced AI methods may not fully capture the complexity of host-associated microbiomes.

### Integrating additional measurements

In addition to microbiome analyses, clinical studies must include comprehensive assessments of host health. Monitoring for adverse events is crucial, but this is often limited to short-term surveillance. It is equally important to implement strategies for long-term monitoring, as potential adverse effects – such as increased risk of immune disorders or cancers – may not manifest until years later. Developing robust, long-term post-trial or post-market surveillance systems such as pharmacovigilance or cosmetovigilance or alternatively population-based studies would help identify these delayed outcomes and ensure the safety of microbiome-based interventions over the lifespan [56]. This should be coupled with measuring biomarkers of immune and inflammatory responses, which can indicate interactions between the microbiota and the host immune system. Other relevant health markers should be included depending on the microbiome in question, such as clinical blood markers and anthropometry measurements for studies on the gut microbiome [128,129]. Laboratory analyses may utilize a range of techniques, from quantitative PCR for quantifying bacterial load to shotgun metagenomic sequencing for taxonomic and functional profiling.

While metagenomics has advanced our understanding of microbiomes, challenges remain. The focus has largely been on taxonomy, but different strains within the same species can have vastly different genomic content, complicating analyses. For instance, *Phocaeicola vulgatus*, a prevalent bacterium in the human gut, has a core genome of 2,257 genes but an accessory genome exceeding 44,000 genes, highlighting the need for strain-level resolution [130]. Additionally, a substantial portion of the genes identified in the human gut microbiome – often reported to range from 25% to as high as 70%, depending on the methods used – lack functional annotations. This gap complicates efforts to link genetic potential with functional outcomes. Moreover, a significant number of annotated genes may have incorrect or incomplete annotations, further challenging the interpretation of microbiome functions and their impacts on host health. Studies also often overlook microbial eukaryotes, archaea and viruses, which also play critical roles in microbiome dynamics and may influence product safety assessments [131,132]. The potential of AI to analyse multi-kingdom interactions, including those involving eukaryotes, archaea and viruses/bacteriophages, could provide deeper insights into microbiome–host interactions and their safety implications [78].

Metabolomics and RNA sequencing are also valuable for understanding microbial and host gene expression and metabolic activity [117,120]. Metabolites produced by the microbiota are also key indicators of microbiome health and function [133]. Although metagenomics primarily reveals the genetic potential of microbiomes, integrating multi-omics approaches (metagenomics, metatranscriptomics, metaproteomics and metabolomics) can provide a more complete picture of the active microbial functions and their effects on the host. This integration is not yet standard practice, even in clinical trials, and is expensive to implement at scale, but it holds promise for uncovering causal relationships between different omics layers and host phenotypes.

**Summary Box 2: Clinical studies and AI**

Despite careful design and execution, clinical studies often face challenges in generalizing findings to broader populations, particularly those with different demographics or health statuses. Another significant challenge is the long-term safety assessment of microbiome interventions, including potential unintended consequences and off-target effects, which are often beyond the scope of typical clinical studies due to practical, ethical and financial constraints.

Optimizing and documenting methodologies for both microbiome and host measurements across studies is crucial for assessing the safety of microbiome perturbations. Rather than rigid standardisation, a flexible and evolving framework is needed to minimize batch effects and facilitate meaningful data aggregation. This approach acknowledges that scientific understanding continues to evolve, and methodologies must be refined accordingly. Cross-comparing results from studies using different approaches will require sophisticated methods to account for variability while leveraging the strengths of diverse datasets. Emerging AI methods hold promise for simplifying and interpreting heterogeneous datasets, but this requires significant investment in data aggregation, annotation and computational resources, and the openness of AI systems is also critical [127].

AI models offer new tools to manage the complexity of large datasets, but improving the explainability and interpretability of AI-generated results remains a critical goal. Rather than rigid standardisation, the focus should be on enhancing transparency and reproducibility in safety assessments, allowing AI to predict outcomes and quantify associated uncertainties and risks, thereby supporting balanced decision-making. Open access to research methods and, where possible, research data, is a critical element of transparency and reproducibility [127,182].

Additionally, experimental data from *in vitro* models could complement clinical studies by providing insights into specific microbiome functions, offering a valuable tool for evaluating potential long-term effects in a controlled environment.

## Part 3: The potential and limitations of *in vitro* models for safety assessment

In the context of microbiome research, where defining a ‘healthy’ or ‘normal’ microbiome remains challenging, safety assessments are often framed by the absence of adverse effects on the host. However, comprehending the effects of microbiome-targeting products and therapies on host responses and biomarkers is crucial for evaluating safety, much like assessments conducted for all new drugs. *In vitro* models, particularly 2D cell culture systems, are commonly used to study host responses to microbial challenges due to their relative simplicity and lower costs, with additional conditions, e.g. saliva-mimicking media or the addition of lipopolysaccharides, allowing more ‘real-world’ simulations of the microbiome and host environments [134,135]. These models allow for the rapid evaluation of endpoints such as inflammatory cytokine production and cell viability [135]. However, while primary human cells from the relevant tissue are often preferred for their ability to closely mimic the behaviour of human tissues, the use of immortalized cell lines can be advantageous in certain experimental contexts. Immortalized cells, which are genetically modified for continuous growth, can help reduce variability and provide more consistent results, depending on the purpose of the experiment [136]. Additionally, using cells from multiple donors helps to account for individual variability, enhancing the relevance of the findings [134,135]. Despite their utility, 2D cell culture models have limitations, including a lack of 3D architecture and a limited duration in culture, which can affect their ability to simulate real tissue interactions. For example, in studies of the skin microbiome, cell culture models may not accurately represent the interactions between skin bacteria and living keratinocytes, as many bacteria primarily interact with the stratum corneum, the outermost layer of the skin [89]. To address these limitations, more complex models that better replicate tissue biology are often necessary (Table 3).

**Table 3.** *In vitro* models for safety and efficacy testing for microbiome-targeting products/therapies

	Main advantage	Main disadvantage	Most relevant measurable safety endpoint
**2D cell culture models**	Ease of use Low cost Relatively rapid assessment Scalable Moderate/high throughput Good reproducibility	Limited time in culture Lack of 3D architecture – does not mimic *in vivo* host–microbiome interactions	Inflammatory cytokine production Cell viability
Primary human cells	May retain behaviour of human cells in tissues	Multiple donors needed to reflect inter-person variability	
Cell lines	Engineered to enable continuous passage Provide consistency across studies Easier to source	Does not necessarily retain the behaviours of human cells in tissues	
**3D models**	3D architecture More tissue level function More representative of microbe-surface interaction (skin)	Higher cost Often require specialized expertise and equipment	Viability Cytokine production Gene expression Structural changes (fluorescence microscopy, electron microscopy, imaging) Can study microbial communities (viability, growth, metabolites, toxins, compositional changes of mixed populations)
3D skin equivalents	Reproducibility Commercially available	Barrier properties do not resemble that of human skin	
*Ex vivo* human skin maintained in organ culture	Gold standard Closer resemblance to barrier properties of real skin maintained (better mimicking of host–microbiome interaction)	Availability	
Organoids	Preserving organ, disease and patient-specific characteristics and enabling repeated experiments	Luminal side of cells oriented towards the centre – obstacle to study host microbiome interaction Not possible to directly co-culture with strictly anaerobic bacteria because it is an aerobic model	Single or multiple omics readout across many different samples
Linearized organoids	Apical (luminal) side accessible Can be co-cultured with bacteria and microbial derived metabolites introduced into the culture media	Not possible to directly co-culture with strictly anaerobic bacteria because it is an aerobic model More expensive than normal 3D organoids	Single or multiple omics readout across samples; TEER (transepithelial electrical resistance) for barrier integrity and imaging
Microfluidic devices	Incorporate organoid cells with other cell types (immune cells and fibroblasts) Can replicate anaerobic conditions (gut)	Infrastructure and cost needs Standardisation is challenging	Multiple omics readouts from the same sample under select conditions
Organ-on-a chip	Reproducible and controllable environment for both host and microbial cells Precise manipulation of microscale fluids, mimicking the physiological environment of the human organs	Different platforms have different attributes Often difficult to choose the most adequate one	Imaging, multi-omics and cytokine measurements

3D models, such as organoids, ‘organ-on-a-chip’ systems and human tissue explants, offer more realistic tissue-level functions and structures. For instance, while keratinocytes in 2D culture cannot fully mimic the conditions bacteria encounter on the skin surface, skin equivalents or *ex vivo* human skin cultures provide a closer approximation [136]. These models are particularly valuable as the barrier properties of artificial skin constructs may not always accurately reflect those of natural human skin, potentially affecting bacterial adhesion and interactions [136,137]. In the study of the gut microbiome, organoids derived from patient tissues or stem cells are increasingly used to investigate host–microbiota interactions [138]. Organoids maintain the structural and functional characteristics of the original tissue, including organ-specific and patient-specific traits. However, traditional 3D organoids present challenges for microbiome studies because their luminal surface faces inwards, complicating access. Techniques have been developed to linearize these organoids into 2D systems, thereby exposing the apical surface for interactions with microbes or microbial metabolites [139]. Nevertheless, maintaining strictly anaerobic conditions typical of the gut environment remains a challenge, though adaptations like hypoxic environments can provide more physiologically relevant conditions [139,140].

Microfluidic devices or ‘organ-on-a-chip’ systems, incorporating organoid-derived cells, offer a sophisticated platform for simulating and precisely controlling the mechanical forces and continuous flow of luminal content characteristic of living tissues [134]. For example, the HuMiX platform can maintain anaerobic conditions, allowing for a co-culture of organoids with microbes under conditions that closely mimic the *in vivo* environment. However, these systems often separate microbial and host cells with a nanoporous membrane, limiting direct contact [141]. While they allow for multiple omics analyses from a single sample, they may not capture all aspects of direct host–microbe interactions [142]. Given that these systems more closely simulate conditions within human organs, they represent excellent platforms for conducting efficacy and safety assessments of microbiome interventions. For example, microfluidic devices may help replicate the dynamic (and shear force and flow) conditions of the oral cavity, offering a more realistic platform for assessing the safety and efficacy of oral care products and microbiome-targeted therapies [143]. However, these advanced models are costly and require specialized expertise and equipment, which can limit their widespread use. A further significant limitation is that, by their very nature, such models can only assess acute or short-term effects, with limited ability to fully assess potential long-term health impacts of microbiome-based interventions. Additionally, validating these models using *in vivo* data may be needed to determine their relevance and accuracy [144,145]. In the USA, the FDA has, in principle, approved the use of organ-on-chip models to replace some animal experiments in drug safety tests before a drug is given to participants in human trials [146].

Recent advances in single-cell and spatial omics have further enhanced our understanding of these complex models by enabling the detailed characterisation of individual cells and their spatial arrangements within organoids. Imaging techniques, both fixed and live, offer crucial insights into co-culture dynamics, while high-throughput systems allow for extensive screening of microbial species or their metabolites [147,148].

**Summary Box 3: Defining relevant endpoints for harmonisation of *in vitro* measurement methods**

*In vitro* models, particularly advanced 3D systems and microfluidic devices, are rapidly evolving and offer powerful tools for studying host-microbiota interactions and assessing safety. However, more research is required to evaluate their representativeness, given the inherent variability of both the host and microbiomes, as well as the wide range of technologies available [183].

One of the key challenges in using *in vitro* techniques for testing the safety of microbiome interventions is the lack of consistency across approaches and methodologies. Despite the availability of diverse test methods, there is no universally recognized international standard for *in vitro* assessment of products or interventions targeting the human microbiome.

Identifying relevant assays and achieving relative harmonisation will only be possible once the biological endpoints for measuring microbiome perturbations are clearly defined. To address these issues, collaboration between the scientific community, industry and regulatory bodies is crucial for defining and validating these endpoints and establishing a harmonized framework. Such guidelines would not only strengthen safety substantiation claims but also enhance regulatory acceptance and consumer confidence.

## Part 4: Prospective: future directions and challenges

Technological advances have significantly enhanced our ability to identify the taxonomic and functional composition of host-associated microbiota. However, linking these microbiota profiles to meaningful host outcomes remains challenging, especially over various timescales. This difficulty is reflected in the frequent use of the term ‘dysbiosis’ without a clear definition and the uncertainty in predicting the biological impact of changes in microbial abundance [60]. Unlike traditional diagnostic microbiology, where detecting a pathogen can be definitive, microbiome research often deals with subtle and complex shifts that are harder to interpret. Furthermore, while optimizing study sampling methods is critical for ensuring data reliability, practical constraints often necessitate a balance between methodological rigour and feasibility. Factors such as sample storage (e.g. home freezing and buffer stabilisation), self-collection kits returned by post, and pre-processing delays can influence microbiome integrity, and ongoing efforts aim to refine protocols that maintain scientific robustness while enabling broader study participation.

When evaluating the potential health implications of consumer products on microbiomes, a range of human-associated microbiota can be involved, including those in the nasopharynx, oral cavity, digestive tract, reproductive tract and skin. Each of these microbiomes is dynamic, influenced by host genetics, lifestyle, hygiene practices, diet and age, plus a plethora of other factors. A more comprehensive understanding of the relationships between microbial composition, including non-bacterial components, and host health could enhance disease therapies and the safety assessment of microbiome-targeting consumer products, whether the effects of these interventions are intentional or incidental.

Despite the growing prevalence of products that alter the human microbiome, there are no globally standardized safety requirements or pathways [110,149], although The European Food Safety Authority (EFSA) has recently acknowledged the role of the microbiome in impacting and refining future risk assessment approaches [150]. Currently, regulatory requirements differ across regions and can be further influenced by the product’s intended purpose (e.g. medicinal vs. dietary supplements), target population and route of administration [151]. While the EFSA has proposed frameworks like the ‘Qualified Presumption of Safety’ to streamline the assessment of certain probiotics, other regions may use different criteria, such as the Generally Recognized As Safe status in the USA [150,152,154].

The traditional microbiological risk assessment approach, which integrates hazard identification, characterisation and exposure assessment, may not fully address the complexities of microbiome-related risks. Defining a ‘healthy microbiome’ and measuring the impact of interventions remain significant challenges [56,150]. There is, therefore, an increasing interest in exploring alternative approaches, such as focusing on functional outcomes (microbial and host) rather than composition, where *in vitro* models may come into play. AI may play a crucial role in identifying patterns within complex microbiome data [127,155], while methodologies from other fields, like next-generation risk assessment principles used in toxicology, could offer new insights. These approaches aim to ensure that changes in the microbiome, whether from consumer products or other sources, do not pose undue risks to consumers. Most recently, an international multidisciplinary expert panel has developed best practice guidelines and a regulatory framework for clinical microbiome testing, aiming to minimize inappropriate use, address current knowledge gaps and ensure evidence-based, safe implementation in clinical medicine [156].

The development and deployment of microbiome-targeting products raise several ethical considerations that need to be addressed to ensure responsible research and application. Some key concerns include informed consent (including scope, risks and potential long-term outcomes), privacy (microbiome data can be highly personal and may reveal sensitive information about an individual’s health, lifestyle and even ancestry), transparency and openness of AI methods, equitable access (as therapies develop, there is a risk they may only be available to certain populations, exacerbating existing health disparities) and the potential for unintended consequences (such as off-target effects or alterations in the microbiome that could affect health in later life). Thus, ensuring comprehensive ethical oversight, including clear guidelines for the development and marketing of microbiome-targeting products, is also a key consideration.

As the field evolves, interdisciplinary collaboration and innovative methodologies will be crucial in advancing our understanding of the human microbiome and its implications for health and safety. This progress will contribute to the establishment of more consistent and comprehensive regulatory frameworks, providing clearer guidance for the development and assessment of microbiome-altering products.

### Conclusion

The workshop which aimed at reviewing the safety assessments of the oral, skin and gut microbiome perturbations highlighted the importance of interdisciplinary collaboration in advancing microbiome research. Key takeaways include the integration of AI for analysing complex microbiome data and predicting interactions, the necessity of well-designed clinical studies with clear endpoints and high-quality sample metadata and the development of innovative *in vitro* models for safety testing. Special attention is required for vulnerable populations, and tailored interventions may mitigate risks in these groups. Public awareness and education are also crucial for fostering informed decision-making and acceptance of microbiome-targeting interventions. Ongoing research is essential for a better understanding and assessment of microbiome–host interactions. As the field progresses, it is indispensable that regulatory frameworks keep up with scientific advancements balancing the needs for harmonisation of microbiome safety assessment approaches and innovation.

## Abbreviations

AI: artificial intelligence
FDA: Food and Drug Administration
FMTs: faecal microbiota transplants
LBPs: live biotherapeutic products
MAGs: metagenome-assembled genomes
OTUs: operational taxonomic units
RCTs: randomized controlled trials
WHO: World Health Organization
